# Giant Abdominal Mass Caused by Disseminated Hepatic Hydatid Disease Leading to Acute Intestinal Obstruction

**DOI:** 10.14309/crj.0000000000002344

**Published:** 2026-10-26

**Authors:** Xuezhi Wei, Fei Chen, Tianlong Su, Narisu Ma, Haifeng Xu, Jinghong Jiao

**Affiliations:** 1 Department of General Surgery, Xilingol League Central Hospital. Xilinhot City, P.R. China; 2 Department of Gastrointestinal Surgery, Daqinglongnan Hospital. Daqing City, P.R. China; 3 Department of Anesthesia, HeXie Hospital of Jixi City. Jixi City, P.R. China; 4 Wulanchabu Medical College, WulanChabu City, P.R. China

**Keywords:** hepatic echinococcosis, giant abdominal mass, dissemination, intestinal obstruction

## Abstract

Cystic echinococcosis (CE), stemming from the larval phase of *Echinococcus granulosus*, poses a grave public health threat in livestock-dense areas. This report details a fatal case of advanced CE in a 47-year-old man presenting with 7-day abdominal distension and obstipation. Abdominal computed tomography revealed multiple cystic lesions inducing small bowel obstruction. Despite supportive care, the patient's status declined, prompting an exploratory laparotomy on December 12, 2023. Postoperative complications, including intra-abdominal hemorrhage and multiorgan failure, led to death on February 8, 2024. These outcomes emphasize CE's severity, and the urgent necessity for enhanced diagnostic and therapeutic approaches against this neglected parasitosis. CE, a significant zoonotic infection caused by the larval stage of the tapeworm *E. granulosus*, poses a severe public health challenge, particularly in regions with extensive livestock farming. This disease is endemic in several Central Asian countries, notably within the northwest territories of China. In addition, high prevalence rates have been documented in other global regions, including parts of South America, Eastern Europe, the eastern and southern Mediterranean, and specific areas of East Africa. CE can lead to a variety of clinical manifestations, including dissemination to various organs, which is often exacerbated in cases of cyst rupture. A 47-year-old man presented with abdominal swelling and complete cessation of bowel movements for 7 days. Initial assessments, comprising abdominal x-rays and CT scans, revealed multiple cystic formations in the liver and abdominal region, indicative of echinococcosis, coupled with a secondary small bowel obstruction. Despite supportive measures like fasting, gastrointestinal decompression, and parenteral nutrition, the patient's symptoms persisted. On December 12, 2023, an exploratory laparotomy revealed widespread echinococcal disease affecting the liver, spleen, and several other organs. This finding necessitated partial resection of the cyst wall and subsequent drainage. Despite the surgical intervention, the patient experienced postoperative complications such as ongoing intra-abdominal hemorrhage and multiorgan failure. These adverse developments led to a reoperation on December 28, 2023. Ultimately, the patient passed away on February 8, 2024, due to these complications. In conclusion, CE is a serious health concern, especially when it spreads and causes severe complications. If left untreated, the disease can result in fatal outcomes. Future research and clinical focus should aim to improve diagnostic methods and treatments for this often-overlooked parasitic disease. Moreover, enhancing awareness among health care professionals is essential to reduce risks and ensure timely, effective treatment.

## INTRODUCTION

Cystic echinococcosis (CE), caused by the larval tapeworm *Echinococcus granulosus*, is a zoonotic infection that affects both humans and livestock.^1^ Echinococcosis is endemic in Central Asian countries with extensive livestock farming systems.^2^ The major morbidity area is northwest in China, including inner Mongolia, Xinjiang province, Tibet.^3^ CE also is highly endemic in central Asia, South America, eastern Europe, the eastern and southern Mediterranean region, and areas of east Africa.^4^ CE can present with dissemination, especially in cases of rupture, and there can also be unexplained dissemination to other organs, including bones, spleen, brain, pericardium, colon, and other organs.^5–9^ There have also been reports of it inducing acute intestinal obstruction. In this case, there was extensive dissemination in the abdominal cavity, and multiple disseminated implants in laparoscopic organs occurred, forming a large cyst that induced acute intestinal obstruction, which is indeed rare. The purpose of this article was to emphasis the fact that hydatid disease can involve any organ of the body and can have an unusually presentation. In serious condition, CE can lead to severe complications after dissemination. If the disease is not treated properly, it is fatal.

## CASE REPORT

A 47-year-old man was admitted due to abdominal distension and pain. He did not defecate for 7 days. The abdominal pain was described as dull and intermittent, without vomiting or passage of flatus or defecation. The patient stated that the abdominal pain and distension persisted without improvement. An abdominal x-ray taken at another hospital indicated mechanical intestinal obstruction.

The patient was admitted to the hospital on December 6, 2023, for additional evaluation and treatment. An abdominal CT scan revealed multiple cystic and cystic-solid masses in the liver and abdominal cavity (Figure 1). These masses compressed the intra-abdominal organs, and some lesions were difficult to differentiate from the right kidney (Figure 1). This raised suspicion of hydatid disease of the liver and secondary small bowel obstruction. The characteristic imaging features of CE allowed for a definitive diagnosis upon admission.

**Figure 1.**
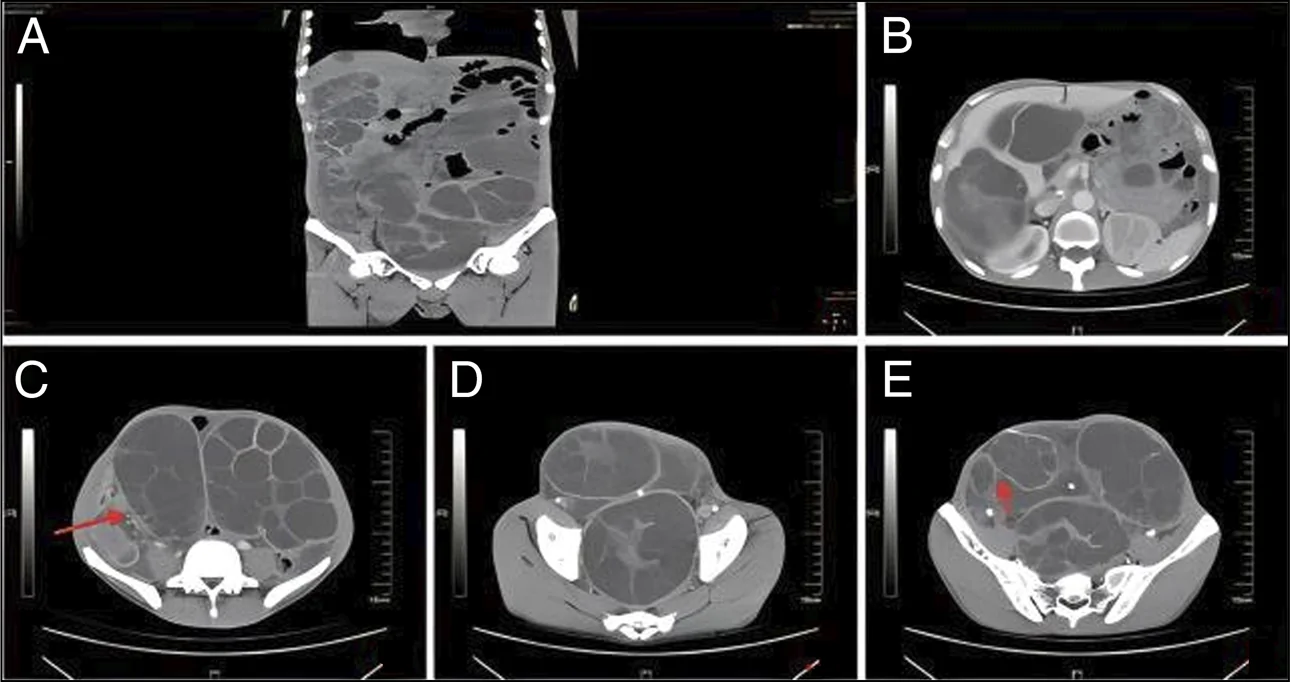
Coronal and axial images of preoperative computed tomography scans. (A; 40×) Coronal view demonstrates hepatic cystic echinococcosis and multiple cystic masses in the abdominal cavity. (B; 40×) The computed tomography axial view indicates hydatid cysts in the liver and gallbladder. (C; 40×) The computed tomography axial view demonstrates hydatid cysts in the appendix and colon. (D; 100×) The axial computed tomography view shows multiple cystic echinococcal lesions in the pelvic region. (E) The computed tomography axial view indicates that multiple hydatid cysts are adhered to the colon.

Upon admission, the patient was kept nil per os (NPO) (nothing by mouth). The patient then underwent gastrointestinal decompression and received total parenteral nutrition support. Despite these interventions, the patient's abdominal distension and pain did not show significant improvement. A follow-up enhanced abdominal CT scan on December 8, 2023, confirmed the presence of multiple cystic and cystic-solid lesions in the liver and spleen, as well as persistent compression of abdominal organs and unclear delineation of some lesions from the right kidney (Figure 1). The findings were consistent with hydatid disease and secondary small bowel obstruction, along with hydronephrosis. Initial laboratory tests, including complete blood count and liver and renal function tests, showed no abnormalities.

The patient resides in a pastoral area and has no significant medical history. On December 12, 2023, the patient underwent exploratory laparotomy under general anesthesia due to unresolved abdominal pain. During the operation, the following findings were observed:

1. Hydatid cysts in the liver (Figure 2).
2. Multiple cystic hydatid lesions throughout the abdominal cavity.
3. Secondary cystic hydatid cysts involving the colon (Figure 2), appendix (Figure 2), gallbladder (Figure 2), and right kidney.
4. Compression of the small intestine with marked adhesions between the bowel and the outer cyst wall.

**Figure 2.**
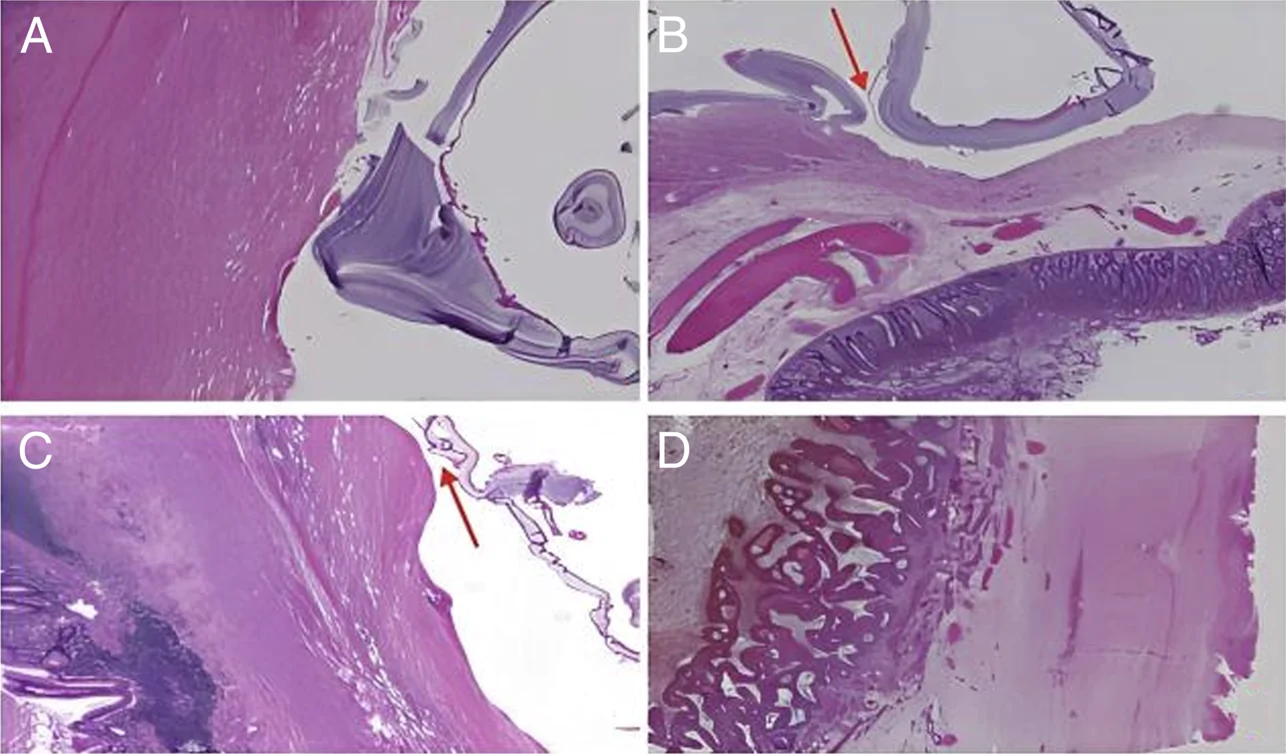
Histopathology image. (A) The postoperative pathology revealed a fibrous cyst wall and an inner laminated membrane characteristic of echinococcosis. (B) The postoperative pathology indicated the presence of a fibrous cystic wall and an inner cyst consistent with colon echinococcosis. (C) The postoperative pathology indicated a fibrous cystic wall and an inner capsule of the appendiceal echinococcosis. (D) The postoperative pathology showed a fibrous cystic wall associated with cystic echinococcosis in the gallbladder.

A partial cyst wall resection was performed, and cystic fluid was aspirated. A 20% hypertonic saline solution was used for hydatid cyst treatment. The outer cyst was incised, and daughter cysts were removed. The outer cyst that infiltrated the appendix was excised, and the outer cyst involving the liver was completely removed, with the liver cut surface sutured. The common bile duct was incised, and saline injection revealed bile leakage from the cut liver surface, which was subsequently sutured. Significant adhesions between the cyst wall and the right kidney necessitated resection of the cyst wall. The kidney cut surface was sutured. Hydatid lesions infiltrating the gallbladder and appendix were removed along with the respective organs. In addition, an internal fistula was found between the sigmoid colon and the cyst wall. Approximately 10 cm of the sigmoid colon was resected and then anastomosed using a linear stapler. Multiple cystic nodules in the omentum were completely excised, numbering in the hundreds.

Biliary drainage tubes and bilateral pelvic drainage tubes were placed before closing the abdominal cavity. Intraoperative blood loss was approximately 600 mL. Postoperatively, the patient received supportive care and oral albendazole (ABZ).

On the fifth postoperative day, the patient presented with hemorrhagic drainage and enteric output from the drainage tubes. Despite receiving symptomatic treatment, including antibiotics and blood transfusions, the patient showed no significant improvement, and bleeding persisted. On December 28, 2023, the patient underwent a second exploratory laparotomy. The surgery revealed small bowel bleeding and fistulas at the ileocecal junction and ascending colon. The fistulas were sutured, and a temporary stoma was created for the ileum. An endoscopic examination of the colonic fistula did not reveal active bleeding, and a stoma was also placed there. A double-lumen stoma was established on the right side, connecting the ileum to the abdominal wall. During the procedure, diffuse bleeding in the small intestine was observed without any obvious bleeding points. Therefore, a stoma was placed for the small intestine.

After surgery, the patient returned to the ward for supportive care. However, the patient developed coagulopathy accompanied by persistent drainage bleeding. Despite receiving multiple large-volume blood transfusions, plasma, and clotting factors, the patient showed no improvement. On February 8, 2024, the patient experienced persistent hypotension and decreased oxygen saturation, resulting in multiple organ failure and death.

The general treatment process comprises the following steps:

Day 1: After admission, symptomatic treatment, antispasmodic and analgesic measures, as well as nutritional support therapy were implemented.

Day 6: Abdominal exploration surgery was performed. Postoperatively, cefoperazone was administered for infection prophylaxis, hemostatic therapy was provided, and nutritional support was continued.

Day 8: Occurrence of hypoproteinemia, supplementation of albumin.

Day 9: There was bleeding from the drainage tube and digestive fluids began to appear on the right side of the drainage.

Day 17: A coagulopathy developed, accompanied by severe anemia. Blood transfusion and plasma infusion were administered.

Day 22: Owing to continuous bleeding and significant loss of digestive fluids, a second surgical intervention was indicated.

Day 17–64: Profuse bleeding from the drainage tube persisted. Daily plasma and leukocyte-depleted red blood cell suspension were administered.

Day 64: The patient developed hemorrhagic shock and exhibited extensive bleeding from the oral mucosa and drainage tube, ultimately succumbing despite resuscitation efforts.

## DISCUSSION

This case is significant because it highlights the complexities involved in diagnosing and managing hepatic echinococcosis that causes secondary small bowel obstruction in a patient with various organ involvement. The unique presentation of multiple cystic lesions compressing adjacent organs, combined with the acute onset of symptoms, underscores the importance of thorough diagnostic imaging and clinical assessment in cases of hepatic echinococcosis with multiorgan involvement.

Reviewing the current literature shows that hepatic echinococcosis predominantly affects the liver.^10^ Complications such as biliary obstruction and secondary infections are well documented.^11^ Despite this, the coexistence of extensive echinococcal disease affecting multiple abdominal organs, as seen in this patient, is less frequently reported. Established hypotheses suggest that such extensive involvement may arise from factors such as parasitic load, host immune response, and anatomical predispositions.^12^ This case adds to the body of evidence indicating that echinococcosis can present atypically, challenging the conventional understanding of its pathophysiology and clinical implications.

The treatment strategy in this case was multifaceted. It involved surgical intervention to manage both the cystic lesions and the resultant bowel obstruction. The necessity for surgery was highlighted by the patient's failure to respond to conservative management, including bowel rest and nutritional support. Surgical resection of the extensive cystic lesions was critical in relieving the obstruction and preventing complications such as bowel necrosis or perforation.^13^ This case clearly demonstrated the inherent risks of extensive surgeries, including significant intraoperative bleeding and postoperative complications. The challenges of postoperative care, such as controlling bleeding and addressing enteric fistulas, reflect the complexities often encountered in extensive echinococcosis cases.

This case clearly reminds us that patients with echinococcosis can deteriorate rapidly, especially when complicated by small bowel obstruction. Multiple organ involvement emphasizes the need for early recognition and intervention. Moreover, the patient's progression to multiorgan failure underscores the importance of careful postoperative monitoring and management to prevent such outcomes.

In conclusion, this case highlights the urgent need to increase awareness and ensure prompt recognition of hepatic echinococcosis and its complications. Future research should focus on improving early diagnostic methods and standardizing treatment protocols to enhance patient outcomes in complicated echinococcosis.^14,15^ Early intervention may significantly alter the disease trajectory, emphasizing the need for continuous education in endemic regions.

## DISCLOSURES

Author contributions: X. Wei and F. Chen designed the study and wrote the paper; T. Su and H. Xu collected the patient's clinical data; N. Ma analyzed the image data; the pictures were supplied by J. Jiao. All authors contributed to the article and approved the submitted version. X. Wei is the article guarantor.

Financial disclosure: None to report.

Informed consent was obtained for this case report.

## ABBREVIATIONS:

ABZ: Albendazole
CE: Cystic echinococcosis
CT: Computed tomography
NPO: Nil per os of interest
